# Breath Biomarkers in Diagnostic Applications

**DOI:** 10.3390/molecules26185514

**Published:** 2021-09-11

**Authors:** Y Lan Pham, Jonathan Beauchamp

**Affiliations:** 1 Department of Sensory Analytics and Technologies, Fraunhofer Institute for Process Engineering and Packaging IVV, Giggenhauser Straße 35, 85354 Freising, Germany; y.lan.pham@ivv.fraunhofer.de; 2 Department of Chemistry and Pharmacy, Chair of Aroma and Smell Research, Friedrich-Alexander-Universität Erlangen-Nürnberg, Henkestraße 9, 91054 Erlangen, Germany

**Keywords:** exhaled breath, breath tests, exhaled biomarkers, metabolomics, clinical practice, volatile organic compounds

## Abstract

The detection of chemical compounds in exhaled human breath presents an opportunity to determine physiological state, diagnose disease or assess environmental exposure. Recent advancements in metabolomics research have led to improved capabilities to explore human metabolic profiles in breath. Despite some notable challenges in sampling and analysis, exhaled breath represents a desirable medium for metabolomics applications, foremost due to its non-invasive, convenient and practically limitless availability. Several breath-based tests that target either endogenous or exogenous gas-phase compounds are currently established and are in practical and/or clinical use. This review outlines the concept of breath analysis in the context of these unique tests and their applications. The respective breath biomarkers targeted in each test are discussed in relation to their physiological production in the human body and the development and implementation of the associated tests. The paper concludes with a brief insight into prospective tests and an outlook of the future direction of breath research.

## 1. Introduction

The human metabolome represents the entirety of low molecular weight molecules present in the human body for a given physiological state and environmental conditions [1]. Metabolomics research undertakes to characterise these metabolic profiles that manifest in relation to health, disease, or environmental burden, with the aim of their prospective use in diagnostic applications or for drug-based interventions [2]. The field has evolved rapidly in recent years, driven foremost by technological advancements in analytical instrumentation (higher resolution/sensitivity, lower detection limits) and the development of sophisticated data treatment tools (multivariate chemometric methods).

Various biological sample types can be used to extract metabolic profiles, with their collection proceeding either invasively, e.g., blood serum or tissue biopsies, or non-invasively, for instance faeces, urine, sputum or breath. Invasive sampling is intrinsically associated with an element of discomfort (that can lead to low patient compliance), limited sample medium and collection frequency (e.g., blood is not exhaustive and can be sampled only intermittently) and typically higher costs. Non-invasive sampling overcomes many of these hurdles, although stool, urine and sputum are not limitless in supply and patient uptake can be compromised due to embarrassment for the former two, and discomfort for the latter. With these considerations in mind, exhaled breath represents an ideal biological fluid for metabolomics research, offering a practically unlimited supply and little to no discomfort to the patient, which encourages cooperation. In addition, exhaled breath can be sampled without the need for privacy or medical personnel and it typically does not generate infectious waste (notwithstanding airborne pathogens [3]), making breath analysis an attractive approach for various applications, from disease detection to exposure assessments [4,5,6,7].

Despite these benefits, the apparent simplicity of breath analysis conceals a complex enterprise. Breath is a rich medium comprising gas-phase inorganic and organic compounds, as well as aerosols in the form of water vapour and particles. Focussing on the gas phase, breath contains several inorganic species and several hundred volatile organic compounds (VOCs) of a diverse chemical nature, the latter being present only in trace quantities [8]. Accordingly, a major challenge in breath analysis is the sensitive detection of individual compounds and the determination of their unique specificity to the disease under scrutiny. The principle reasons for the hitherto moderate pace in progress of breath biomarker discoveries lies in the technological limitations associated with reliably capturing breath and the analytical intricacy of extracting potential biomarkers from complex datasets [9,10]. Moreover, the lack of standardisation in breath analysis has led to a limited alignment of results between independent studies employing different approaches [11]. The need to develop and introduce standardised practices in breath research is widely accepted, and adopting such measures is expected to expedite progress in the field [11,12,13], as has been demonstrated in the successful development and implementation of the nitric oxide breath test for asthma, as discussed below [14].

Overall, despite the aforementioned limitations and challenges, breath-based metabolomics has been successfully implemented for specific applications and shows promise as a complementary approach to traditional diagnostic tests for characterising health disorders, infections and environmental exposure [15]. This review outlines the case for using breath analysis as a rapid and practical tool in metabolomics research and presents and discusses the gas-phase breath-based tests that have been established and are in (routine) use. The paper commences with a general overview of relevant tests, then divides these topically between tests using compounds of either endogenous or exogenous origin, such as exhaled nitric oxide or breath ethanol, respectively. Each breath test is reviewed in relation to the discovery of the respective biomarker, the associated biochemical or physiological processes of biomarker production and/or metabolism in the human body and its subsequent excretion via breath, and ultimately the practical or clinical implementation of the test. An in-depth discussion of the benefits and limitations of breath analysis and a critical discourse of the different sampling and analytical approaches have been reviewed at length in the scientific literature [4,10,16,17,18]; thus, they are not treated here. Similarly, this review focusses on the compounds present in the gas phase of exhaled breath; the tests based on exhaled breath condensate (EBC), aerosols (EBA) or particles are not covered in this paper. 

## 2. Approved and/or Established Breath-Based Tests

The volatile (gas-phase) fraction of breath comprises compounds of different origins, which are broadly categorised as endogenous or exogenous. Endogenous compounds derive from the host metabolism, particularly from routine metabolic processes, but also from an imbalance in the body relating to disease or (organ) dysfunction [19]. These compounds reflect the momentary physiological state of an individual and are of potential interest and use as biomarkers [20]. The regular metabolism in a healthy individual drives the production, distribution and fate of endogenous compounds. When adverse changes in the body up- or downregulate these processes, the associated changes in compounds prospectively manifest in exhaled breath [21,22]. In contrast, exogenous VOCs represent compounds from the environment. These are ubiquitous, and their intake into the body proceeds either through the inhalation of ambient constituents and pollutants, via the ingestion of food or drugs, or by dermal absorption [8,17]. A large proportion of the VOCs hitherto detected in exhaled breath are—or derive from—exogenous compounds [8,9,23]. An additional source of origin of VOCs, referred to as biological/non-host [17], is represented by the microbiome [24,25,26]. The interaction of the host and commensal microorganisms, especially in the human gut microflora, contributes significant complexity to the human metabolome [27], whereas the metabolism of the lung (and oral/nasal) microbiome may also affect the exhaled VOC profile [28]. The VOCs metabolised or produced in vivo by bacteria are of clinical interest and potentially provide indications of pathogen infections [24]. The symbiotic nature of the microbiome raises the question as to whether these bacteria should be considered a part of the human organism or rather treated as separate entities; accordingly, there is no current consensus on how the compounds associated with these organisms should be designated, either as endogenous or as exogenous, hence the additional ‘biological’ categorisation.

From the considerations above it is clearly evident that the origin of any individual compound present in breath is rarely distinctive. Human breath composition has manifold contributing factors; therefore, determining the specific origins of individual biomarkers is challenging due to multiple potential sources [20,24]. Despite these difficulties, breath research has made steady advances in health-related applications in its relatively short recent history. Several breath-based tests have met with medical or regulatory approval, such as through the United States Food and Drug Administration (US FDA) or the European Union European Medicines Association (EU EMA), or are endorsed and/or recommended by professional associations and are now in regular or routine use [29]. These approved and established tests, which are discussed individually in the ensuing sections of this review, are summarised in Table 1.

## 3. Tests Targeting Endogenous Compounds

The endogenous compounds present in breath derive from regular internal metabolic production, with either subsequent systemic circulation and transition into the gas-phase after passing the alveolar-blood capillary membranes in the lungs, or direct release into exhaled gas in the case of localised airways production [19,30]. A variation in the volatile composition of exhaled breath can provide insights into the corresponding (adverse) changes in the body [20]. As such, the majority of breath research hitherto undertaken has focused on exploring endogenous disease biomarkers. 

The applications of endogenous compound-based breath tests that are currently implemented range from widespread routine use to highly specific settings. The most common test uses carbon dioxide, a by-product of cellular respiration, as a breath-borne marker to monitor breathing (e.g., in intensive care or sedated patients), whereas other tests target specific compounds for particular cases, e.g., nitric oxide in patients with asthma, or carbon monoxide in infants at risk of neonatal jaundice, demonstrating the prospects of breath tests based on a symptomatic approach. The Heartsbreath test for heart allograft rejection further shows the applicability of breath testing of multiple compounds as a screening procedure for adverse events. This section reviews the breath tests that utilise endogenous compounds, starting with the most common and widely implemented procedure.

### 3.1. Capnography

**Figure 1:**
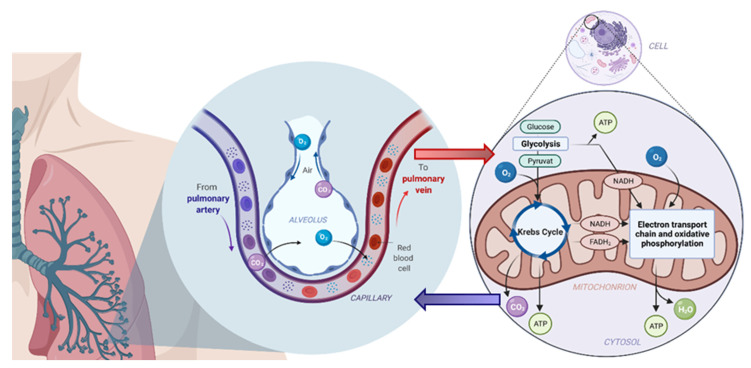
Exchange of respiratory gases—oxygen (O_2_) and carbon dioxide (CO_2_)—within the alveoli during aerobic cell respiration; the process of aerobic cell respiration and CO_2_ production is depicted on the right: pyruvate, generated from glycolysis of glucose, and O_2_ enter the Krebs cycle to form CO_2_ and produce the energy-intermediate adenosine triphosphate (ATP), as well as the nicotinamide adenine dinucleotide anion (NADH) and the hydroquinone form of flavin adenine dinucleotide (FADH_2_). During oxidative phosphorylation, electrons are transferred from NADH and FADH_2_ to O_2_ by a series of electron carriers to form ATP and water (H_2_O). Created with BioRender.com.

The practice of measuring carbon dioxide (CO_2_) during respiration is known as capnography. Specifically, capnography refers to the continuous analysis of CO_2_ partial pressure in respiratory gas [31]. The widespread, routine implementation of capnography throughout the world makes CO_2_ the most widely exploited and commonly used breath biomarker. Research on CO_2_ in exhaled breath boasts an early history, with its presence in breath first discovered in 1784 by Lavoisier and Laplace through their investigations of breath from guinea pigs. Antoine Laurent Lavoisier (1743–1794) and Pierre Simon Laplace (1749–1827) devised a system that enabled pre-concentrated breath to be purged through a device containing a chemical solution that reacted with CO_2_ in the gas to form a precipitate and indicate its presence. Accordingly, these experiments allowed Lavoisier and Laplace to demonstrate for the first time that CO_2_ is a constituent of exhaled breath, which they associated with the respiratory metabolism (Figure 1) [32]. 

During the same period, researchers began to study the association between CO_2_ and plant life, which ultimately led to the development of the theory of photosynthesis in the 1770s by the physiologist Jan Ingen-Housz (1730–1799) [33]. Photosynthesis can be essentially considered the reverse of respiration that acts as a mechanism for plants to store energy, with an uptake of CO_2_ and the release of oxygen (O_2_) following a series of reactions. In 1803, John Dalton (1766–1844) posited that the molecular structure of CO_2_ consisted of one carbon atom and two oxygen atoms. Due to the asymmetric and polyatomic nature of CO_2_, it strongly absorbs light with wavelengths in the infrared (IR) spectrum, a phenomenon that was exploited in one of the earliest IR measurements of CO_2_ in breath when John Tyndall (1820–1893) constructed an apparatus that measured the absorption of various gases and vapours, including CO_2_ [34]. 

The physiological production of CO_2_ is now well understood. The process of aerobic cell respiration involves a series of metabolic reactions that breakdown glucose to CO_2_ and water (H_2_O) and generate the energy-intermediate adenosine triphosphate (ATP). In the presence of O_2_, pyruvate, which is an intermediate product of glycolysis, is oxidised and enters the Krebs cycle (citric acid cycle) as acetyl coenzyme A (acetyl-CoA), whereupon metabolic transformations lead to the release of CO_2_ as a by-product (Figure 1, right-hand side) [35], which is then transported via the bloodstream into the lungs and is ultimately expelled from the body in exhaled breath (Figure 1, left-hand side).

**Figure 2:**
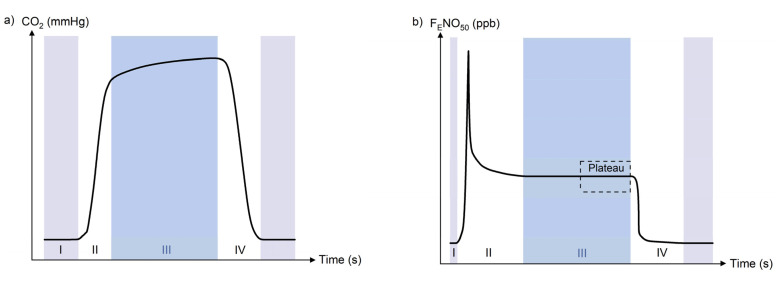
Schematic depictions of (**a**) the respiration cycle as described in relation to carbon dioxide (CO_2_) partial pressure—referred to as a capnogram—comprising four transitional phases, namely: phase I, end of inspiration and dead-space gas; phase II, mixed-airway and alveolar gas; phase III, end-tidal volume; and phase IV, inspiration; note that CO_2_ is offset from the vertical axis for a clearer depiction (figure adapted from [50]); and (**b**) fraction of exhaled nitric oxide (F_E_NO) during respiration through the mouth at a flow rate of 50 mL/s, with depicted phases representing: phase I, inspiration with air; phase II, mixed-expiratory gas, including the initial peak of exhaled NO after inhalation via the nose (from local nasal NO production); phase III, steady flow region (the 3 second plateau from which the F_E_NO value is extracted is indicated); and phase IV, inspiration (figure adapted from [51]).

The clinical exploitation of exhaled CO_2_ in the form of capnography has been widely implemented for over three decades, being a staple procedure in clinical practice since the late 1980s [36,37]. Besides its basic assessment of lung ventilation, capnography has become an important component during general anaesthesia, conscious sedation, intubation, patient movement or transportation and other procedures, providing an essential indicator for patient safety and offering valuable physiological data on ventilation and perfusion matching in the lungs, cardiac output and metabolic rate [38,39]. A schematic figure of the characteristic CO_2_ partial pressure in breath during respiration, referred to as a capnogram, is depicted in Figure 2a.

Generally, instrumentation for capnography operates based on one of two technologies, either colorimetry or infrared sensors. Colorimetric CO_2_ devices utilise a pH-sensitive material that changes colour in relation to the amount of CO_2_ present, thereby offering a qualitative and semi-quantitative detection of CO_2_. Colorimetric detectors are portable and disposable, yet are prone to false positive readings through pH-influencing contaminants [40,41]. Accordingly, the use of IR sensor-based technologies is the preferred choice for both intubated and spontaneously breathing patients. As a spectroscopic method, IR-based systems detect the presence of CO_2_ through absorption bands in the electromagnetic spectrum and the CO_2_ waveform is subsequently displayed as a function of time or exhaled volume [31,38]. The compact nature of IR sensors has led to the development of palm-sized miniature CO_2_ monitors [42,43], making bedside observation practical, as well as being particularly tolerable for vulnerable patient groups, such as children or the elderly [44]. IR sensors are sensitive and accurate, although they are susceptible to damage when handled or through fouling due to expectorate secretions and circuit condensate [31]. Monitoring the CO_2_ in breath can be performed via mainstream or sidestream sampling. The former relates to the direct analysis of the entire breath fraction through the inline positioning of the capnograph, whereas the latter samples respiratory gas through a side port of the breathing circuit (mainly for intubated patients), and as a consequence, incurs a time lag of several seconds before displaying the results [31,45].

Despite its benefits and widespread use, capnography is not universally implemented in medical settings owing to a limited availability and accessibility of equipment in some areas, disparate views of medical personnel on its ability to impact or improve patient care, and the contentious interpretation of capnograms. Accordingly, exhaled CO_2_ monitoring is often underutilised, despite its high potential in ensuring patient safety [36,46,47,48]. Nevertheless, the importance and acceptance of capnography in the clinical setting is evident by the numerous standards, guidelines and advisory statements, as well as its recognition by several professional institutions and regulatory agencies, including the American Society for Anesthesia, the American Heart Association, the American Association for Respiratory Care, the Joint Commission and the Centers for Medicare and Medicaid Services, and the Association of Anaesthetists of Great Britain and Ireland, amongst others [37,49]. Based on the evidence of its efficacy in medical care and the overwhelming consensus of medical practitioners, capnography represents an important procedure to monitor patient stability, thus exhaled CO_2_ can be considered a key and widely exploited breath-borne biomarker in healthcare.

### 3.2. Nitric Oxide Breath Test for Asthma

Nitric oxide (NO) in exhaled breath has been successfully exploited as a biomarker for asthma screening and treatment management since the turn of the millennium. Following discrepancies in the observed concentrations of exhaled NO between early studies, it soon became apparent that the fraction of exhaled nitric oxide (F_E_NO) exhibits a flow dependency [52,53], thus must be measured under defined and well-controlled conditions to generate reliable data to indicate inflammation of the airways [54]. These findings led to the development of comprehensive guidelines for accurate F_E_NO detection that were indispensable for establishing a reliable breath test for asthma.

The history of utilising F_E_NO measurements in healthcare dates back approximately two decades, yet the history of NO research extends back further. NO is an inorganic molecule that was first discovered in 1774 by the British chemist—and contemporary of Lavoisier—Joseph Priestley (1733–1804) [55]. Early research on NO in the last century focussed on its role in atmospheric chemistry, notably in the production of tropospheric ozone and its contribution to photochemical smog, and two centuries were to transpire since its discovery before an association to human physiology was made. In the late 1970s and early 1980s, independent physiological and pharmacological studies by Ferid Murad, Robert Furchgott and Louis Ignarro on vasodilation, mechanisms and modes of action of vasodilating and muscle relaxing drugs, and the component termed ‘endothelial-derived relaxing factor’ (EDRF), led to the discovery of NO as an endogenous mediator in health and disease (this work was later acknowledged with the Nobel Prize in Physiology or Medicine for the ‘discoveries concerning nitric oxide as a signalling molecule in the cardiovascular system’, awarded to the trio in 1998) [56,57,58]. The presence of NO in mammalian exhaled breath was first reported in 1991 in a seminal paper by Lars Gustafsson and colleagues, who demonstrated its endogenous production by NO synthase through the use of enzyme inhibitors [59,60]. This discovery was soon followed by evidence that the NO in exhaled breath was present at higher concentrations in asthmatics [61,62,63], which opened the path for the use of NO as a breath-borne biomarker. Another notable discovery was made in 1995, namely that epithelial cells in the paranasal sinuses produced NO at high concentrations [64], highlighting a potential confounding contribution to F_E_NO from nasal air. 

**Figure 3:**
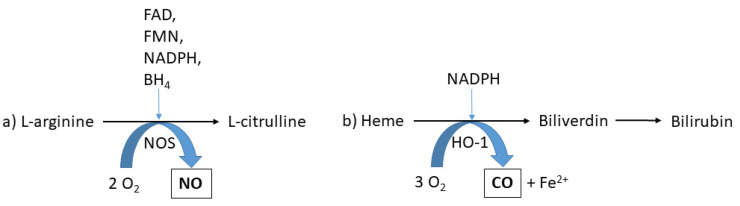
(**a**) Synthesis of nitric oxide (NO) from l-arginine in the presence of oxygen (O_2_), with nitric oxide synthases (NOS) and the cofactors reduced nicotinamide adenine dinucleotide phosphate (NADPH), flavin adenine dinucleotide (FAD), flavin mononucleotide (FMN), and tetrahydrobiopterin (BH_4_); (**b**) Heme degradation pathway: heme metabolism by heme oxygenase enzymes requires O_2_ and NADPH (with NADPH CYP450 reductase) with heme oxygenase-1 (HO-1) to generate equimolar carbon monoxide (CO), iron (Fe^2+^) and biliverdin, which is reduced to bilirubin by NAD(P)H biliverdin reductase.

Nitric oxide is produced in the body by a group of enzymes, nitric oxide synthases (NOS), when l-arginine is oxidised to l-citrulline in the presence of oxygen and cofactors (Figure 3a) [65,66,67]. NO has diverse functions in blood vessels and the airways, such as for smooth muscle relaxation and vasodilatation for matching regional airflow and blood flow [68]. In the respiratory system, NO is produced in the lung alveoli, proximal and upper airways, as well as in the nasal cavity, diffusing over cell membranes via a concentration gradient and released into the airways [69]. The elevated levels of NO in asthmatics are associated with inducible NOS (iNOS), which is a variant of NOS that is expressed when primed by inflammatory stimuli [70,71] but exhibits inhibitory effects after the administration of corticosteroids [65,72].

**Figure 4:**
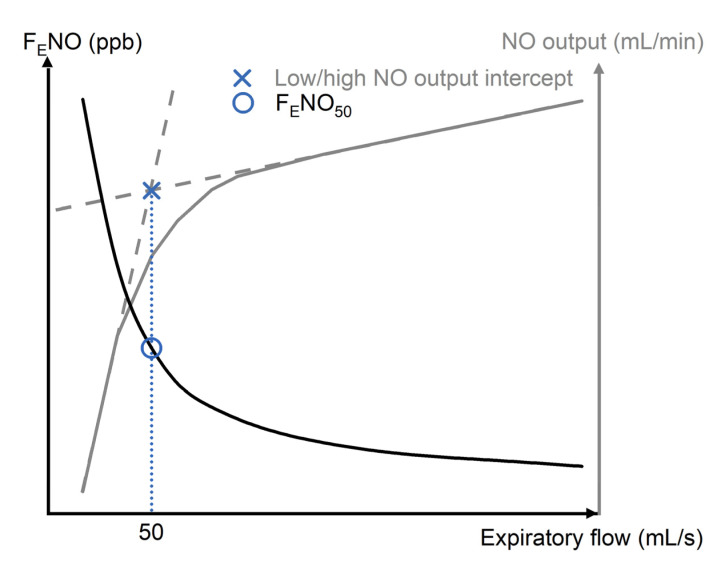
The fraction of exhaled of nitric oxide (F_E_NO) and NO output as a function of expiratory flow, whereby 50 mL/s is recommended as the standardised flow for the F_E_NO breath test (specified as F_E_NO_50_; see text). The F_E_NO value is derived from the intercept of the regression lines for low and high NO output, as illustrated. (Figure adapted from [79]).

The observations in the early 1990s that airway inflammation associated with asthma leads to an increase in F_E_NO, and that an inhibition of iNOS via inhaled corticosteroids (ICS) treatment acts to lower F_E_NO levels, soon placed exhaled NO as a promising inflammatory marker of asthma [69,73,74]. Several discoveries soon followed, including observations of reduced F_E_NO levels in smokers [63,75], age-dependent concentrations in children [76], and the influence of exogenous factors, such as caffeine ingestion, on F_E_NO levels [77]. Notably, the F_E_NO concentration was found to depend on exhalation flow, whereby levels were observed to decrease at higher flow rates (Figure 4), thus there was an immediate need to establish standardised practices to ensure accurate and reliable readings [14]. This phenomenon can be explained by the flow-dependency of NO output, where the exhaled gas at low flow will have time to be enriched with NO from the upper airways, whereas the alveolar gas constitutes a large part of F_E_NO at high flow; the linear portions of NO output at low and high flow intercept at an expiratory flow of around 50 mL/s, regardless of the F_E_NO concentration; this is designated with the term F_E_NO_50_ (Figure 4) [78].

To address this aspect, the European Respiratory Society (ERS) created a taskforce to gather and provide consensus recommendations amongst experts, which was published in 1997 [80], followed shortly afterwards by similar guidelines compiled by the American Thoracic Society (ATS) [81]; a joint guidelines paper of the two societies containing revised recommendations for standardised methods of measuring and reporting exhaled NO was published in 2005 [82]. These guideline documents were decisive in aligning practices on NO measurements (including specifying an expiratory flow of 50 mL/s) and in generating an understanding of NO in the human physiology, and therefore, played a pivotal role in establishing the F_E_NO breath test for asthma. The concurrent development of a chemiluminescence-based NO analyser specifically for F_E_NO measurements in breath resulted in the launch of the commercial NIOX^®^ device by Aerocrine AB (Solna, Sweden), which received an FDA approval for its use in detecting and monitoring asthma in 2003 [29,83]. 

Measurement scenarios for F_E_NO differ depending on the application and setting. Apart from offline methods in which exhaled breath is first collected in a reservoir for subsequent analysis, as often implemented in large-scale studies [84,85,86], online NO measurements use an NO analyser for direct sampling and immediate analysis [87,88,89]. Online measurements may result in higher data quality, whereas offline testing can be more practical in some scenarios [90]. F_E_NO sampling can be achieved either through continuous tidal breathing or single-exhalation episodes, with the latter generally being the favourable approach. Due to the localised generation of NO in the nose, sampling nasal air should be avoided [65,91]. The standardised single-breath technique specifies that a patient should inhale to total lung capacity (TLC), then exhale at a constant flow rate of 50 mL/s against resistance (to exclude nasal NO by means of velum closure); the 3-second NO plateau at the end of the exhalation is designated as F_E_NO (Figure 2b) [82]. If inhalation to TLC is difficult to perform, e.g., in patients with severe lung illnesses, small children or the elderly, non-TLC deep inhalation may be performed, albeit for an extended period in order to reach the plateau [78]; this alternative manoeuvre is more comfortable for the patient and has been adopted by clinicians, as well as in instrument designs of manufacturers [92]. Ventilated patients pose another challenge for single-breath measurements, with studies describing alternative methodologies for intubated subjects [93].

Nowadays, the most common methods for NO measurement are chemiluminescence, electrochemical sensors and laser-based technology, with chemiluminescence representing the gold standard due to its high sensitivity, low detection threshold (ppb level) and fast response time (0.5–0.7 s) [94]. F_E_NO values can be influenced by several non-disease-related factors, such as genetics, sex, weight and height, diet (e.g., caffeine intake), drug use or smoking, thus a questionnaire accompanying NO measurement is recommended to establish the potential confounders. Due to this variability, the Global Initiative for Asthma (GINA) recommends the use of additional parameters for asthma screening rather than a diagnosis based solely on F_E_NO, although the guidelines nevertheless recognise that the use of F_E_NO for treatment can lead to fewer exacerbations compared to treatment based on the current guidelines [94,95]. Overall, the clinical utility and diagnostic value of F_E_NO for asthma management exhibits a sensitivity that ranges between 79% and 86% and specificities between 85% and 89% [96].

Presently, F_E_NO is becoming increasingly established in the general clinical guidelines for asthma diagnosis. The British National Institute for Health and Care Excellence and the Scottish guidelines for asthma diagnosis and management, for instance, recommend F_E_NO testing in combination with other diagnostic tests to help diagnose asthma or support asthma management in symptomatic patients despite ICS treatment [94]. Today, F_E_NO is used to predict and monitor the ICS response [97,98] and adherence [99,100], and to diagnose ICS-naive patients [95]. Overall, the F_E_NO breath test for asthma diagnosis demonstrates the high potential for breath-based diagnostics, with NO representing the most successful breath-borne biomarker for disease indication; it should be stressed again here that this success is directly attributable to the exhaustive studies on F_E_NO in relation to sampling and analysis and the ensuing extensive guidelines on the related required practices.

### 3.3. Exhaled Carbon Monoxide in Neonatal Jaundice

Carbon monoxide (CO), an inorganic gaseous molecule primarily associated with the combustion of fossil fuels or organic matter, was first discovered in the late eighteenth century in the era of Lavoisier and Priestley. Indeed, it was Priestley who first produced CO in 1772 through heating charcoal, which he termed ‘combined fixed air’ [55]. The presence of CO in blood was first demonstrated in studies at the turn of the twentieth century [101,102], followed by observations in 1949 of its endogenous production, which was associated with bilirubin production [103]. The predominant source of endogenous CO is heme degradation by heme oxygenase (HO) enzymes (EC: 1.14.14.18) [104,105,106]. This enzymatic oxidation is catalysed primarily by heme oxygenase-1 (HO-1), the inducible form of HO, though the constitutively expressed isozyme HO-2 may also contribute [107]. Subsequently, CO binds with high affinity to haemoglobin to form carboxyhaemoglobin, which is transported through the systemic circulation to the lungs, where CO transitions into the gas-phase at the alveoli and is then excreted from the body via exhaled breath (Figure 3b) [108]. Besides CO, heme catabolism during red blood cell turnover in normal human physiology produces bilirubin [109,110]. The accumulation of bilirubin in neonates through elevated HO activity leads to hyperbilirubinemia, commonly known as jaundice, which causes a yellow-orange pigmentation of the skin, sclerae and other tissues [110]. Bilirubin production in normal term neonates is known to be two to three times higher than in adults [111]. Due to the neurotoxic nature of bilirubin, severe jaundice, caused by its accumulation to excessively high concentrations, is considered a pathophysiological condition, causing permanent neuronal damage, and is referred to as bilirubin-induced neurologic dysfunction (BIND), or kernicterus [112,113,114,115]. 

Bilirubin accumulation is exacerbated by haemolysis for a variety of reasons, such as the breakdown of extravascular blood, maternal diabetes or infant prematurity, as well as hitherto unknown causes associated with ethnicity, and other conditions [111]. Accordingly, current preventive or therapeutic measures for severe hyperbilirubinemia, such as phototherapy or blood exchange transfusions, are nonspecific [110]. The concomitant production of CO with bilirubin during heme degradation and its subsequent excretion via breath have been studied extensively. Due to its stoichiometry, the measurement of CO in breath can be used effectively as an index of bilirubin accumulation and thus, hyperbilirubinemia in vivo [109,116]. Specifically, it has been shown that the (pulmonary) excretion rate of CO (VeCO) represents a reliable measure to estimate bilirubin accumulation. Early studies of CO in the exhaled breath of neonates made use of an incubator, whereby the airtight hood was fitted with a control module and a gas chromatography (GC) system equipped with a gas detector that allowed the infant’s VeCO to be measured over a 20–30 min period after an equilibration period of 40 min [116]. In 1984, however, a study reported a correlation between VeCO and end-tidal CO (ETCO) in infants without pulmonary disease and, therefore, demonstrated the possibility to use ETCO instead of VeCO as a measure of bilirubin accumulation, offering significant advantages in simplicity and rapidity over the VeCO-based approach [117]. The measurement of ETCO as a marker of haemolysis was traditionally performed using GC [118], but this has transitioned to the contemporary use of more portable and non-invasive systems, such as the CoSense^®^ CO monitor (Capnia, Inc., Redwood City, CA, USA), which exhibit sufficient accuracy and precision for the detection of haemolysis in neonates. Studies on ETCO have shown that concentrations ≥ 2.5 ppm (corrected for ambient CO) are indicative of the presence of significant haemolysis [119,120,121,122], and that this breath-based test exhibits a higher accuracy compared to the direct anti-globulin test (DAT) [123]. Moreover, a study from 2001 found a correlation between the ETCO level associated with neonatal jaundice, even in infants without haemolytic diseases, with high sensitivity, specificity and negative predictive values (86, 80 and 97%, respectively) [109]. Accordingly, this approach has been demonstrated to be an accurate method for the early identification of haemolysis in new-born individuals that offers the opportunity for the timely initiation of phototherapy, thereby reducing the risk of readmission, neuronal toxicity and kernicterus [124]. The efficacy of the exhaled CO breath test for neonatal jaundice in infants led to the procedure receiving an FDA waiver in 2018. Although not in widespread use, this breath-based test represents an important non-invasive approach for screening the most vulnerable members of society that allows for early action in treating this potentially fatal condition. 

### 3.4. Heartsbreath Test in Cardiac Transplant Rejection

Cardiac transplantation is a life-saving treatment for patients with severe heart conditions and heart failure. Despite the scarcity of universal epidemiological data on heart failure, population-based studies have shown representative trends in recent years. The steady increase in admissions of patients suffering congestive cardiac failure has been linked to an ageing population and an increasing prevalence of obesity in younger adults, indicating the growing importance and necessity of transplantation in severe cases [125]. Following the surgical procedure itself, heart transplantation suffers from a high incidence of allograft rejection, despite progress in immunosuppressive therapy, and this represents a leading cause of death in these patient groups [126,127]. Post-surgery, recipients must undertake regular screenings for signs of organ rejection, but associated symptoms, including fatigue, malaise and oedema, amongst others, are uncommon, making rejection episodes difficult to detect [128]. Currently, an endomyocardial biopsy is the gold standard for the diagnosis of heart transplant rejection; this is an invasive procedure that is performed weekly for the first six weeks following allograft surgery, then periodically for at least one year post-surgery [129,130]. The biopsy procedure is itself associated with health risks, especially in immunosuppressed patients, following the original surgery, and oftentimes biopsy results are unremarkable and do not lead to an alteration in patient treatment, thus their benefit is questionable [126,128]. 

Non-invasive alternatives to screen for heart allograft rejection have been increasingly studied over the last few decades, including antibody tests, magnetic resonance imaging, echocardiography and the use of serum markers [131,132], yet these approaches pose deficiencies in their respective accuracy, making them unsuitable for personalised medicine. In the early 2000s, contemporary breath research pioneer Michael Phillips explored the potential to exploit the oxidative stress associated with organ transplantation and the ensuing manifestation of alkanes in exhaled breath [128,133]. Allograft rejection is accompanied by oxidative stress due to the increased production of reactive oxygen species (ROS), which degrade the membrane polyunsaturated fatty acids (PUFAs) caused by free-radical induced lipid peroxidation and ultimately lead to the generation of alkanes and methylalkanes, with their subsequent transition into breath [83,128,134]. Based on this premise, a model was developed that utilises a combination of nine C_4_-C_20_ alkanes and mono-methylated alkanes—termed the breath methylated alkane contour (BMAC)—as markers of oxidative stress [135]. The breath test is accomplished by capturing exhaled VOCs onto a sorbent trap using a portable breath collection apparatus (BCA, Breath Meter Technology, Inc., Cleveland, OH, USA) and subsequently analysing the sample through thermal desorption and gas chromatography-mass spectrometry (TD-GC-MS) [136]. The studies observed an increase in the BMAC in heart transplant patients with grade 0, 1 or 2 rejection, which is attributed to increased myocardial oxidative stress in the heart in the period between the post-mortem removal from the donor and the subsequent allograft in the recipient [137]. In contrast, a paradoxical contour reversal of the BMAC markers observed in patients with grade 3 rejection was observed, indicating that the abundance of these markers decreased in this group. It is hypothesised that this phenomenon is associated with self-induced catabolic processes, in which increased levels of alkanes trigger higher activity in the inducible cytochrome P450 (CYP450) enzymes that deplete the oxidative stress markers in these patients [128]. Overall, this breath-based test for heart transplant rejection—named Heartsbreath—is able to predict grade 3 organ rejection with high accuracy compared to the concordant set of International Society for Heart and Lung Transplantation (ISHLT) grades in biopsies (sensitivity 78.6%, specificity 62.4%, positive predictive value 5.6%, negative predictive value 97.2%) [128,133]. A particular benefit of the Hearthsbreath screening test is that the high negative predictive value allows rejection episodes to be largely ruled out, thereby reducing the need for burdensome biopsies. The Heartsbreath test, developed by Menssana Research (Newark, NJ, USA), received FDA approval through a humanitarian device exemption in 2004, which caters for devices intended to benefit individuals with diseases or conditions affecting fewer than 8000 patients annually in the US [138]. Despite FDA approval, however, the test is currently not in use because it is not covered in the public or private medical insurance programmes in the USA.

## 4. Tests Exploiting Exogenous Compounds

The exogenous compounds in exhaled breath represent substances that originate exterior to—and are taken up by—the body, which are subsequently eliminated via respiration. The primary sources include constituents of ambient air that are inhaled and food-derived compounds or medication ingredients that are ingested or administered. In breath analysis, xenobiotic compounds, i.e., chemical compounds that are foreign to the human organism, have been largely considered as confounding factors in the search for endogenous biomarker compounds [139]. Indeed, a persistent challenge in breath biomarker discovery research has been how to identify these compounds and exclude them from datasets [11], although in certain scenarios these can be utilised to explore aspects relating to the human exposome [140]. In terms of the inhaled compounds, the collection of ambient air samples concurrent to breath sampling provides a baseline dataset of environment constituents that can be used retrospectively to assess potential confounding VOCs in exhaled breath [20,141,142]. Such measures are particularly important in some settings, such as the clinical environment in which the abundant and persistent use of detergents and disinfectants creates a high background of related compounds [143]. An important point to note, however, is that the fate of a VOC of exogenous origin varies according to multiple factors, including the nature of the compound (lipophilicity, volatility), the degree to which it can be metabolised, as well as the concentration and duration of exposure. Consequently, collecting and analysing an ambient air sample at the time of the breath test represents only a snapshot of the immediate exposure but will not throw light on prior exposure, nor will it identify the metabolic by-products of inhaled compounds. In view of the latter, however, this process can be exploited in a targeted way, as discussed below. 

**Figure 5:**
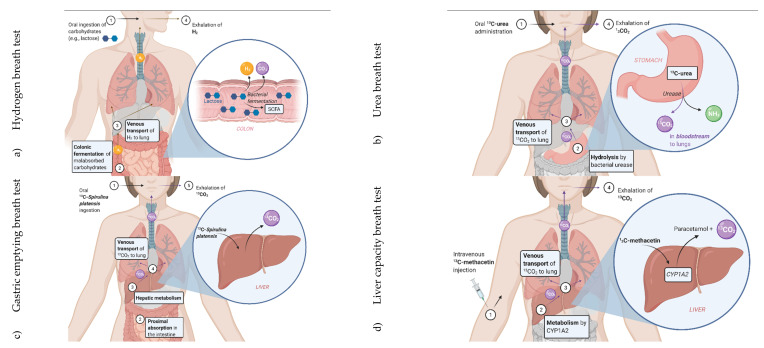
(**a**) Assessment of hypolactasia via the hydrogen breath test (HBT): colonic fermentation of ingested lactose after malabsorption leads to the production of molecular hydrogen (H_2_), carbon dioxide (CO_2_) and short-chain fatty acids (SCFA): the detection of H_2_ in exhaled breath is possible after its venous transport to the lungs; (**b**) Diagnosis of *H. pylori* infection via the ^13^C-urea breath test (UBT): orally ingested ^13^C-urea is metabolised by urease enzyme to form ^13^CO_2_ (and ammonia, NH_3_) in the stomach, which is transported to the lungs and can be detected in exhaled breath; (**c**) Stable isotope breath test for assessing solid gastric emptying using ^13^C-*S. platensis*: the ingested substrate is absorbed in the small intestine and metabolised to ^13^CO_2_ in the liver, which is transported to the lungs and can be detected in exhaled breath; (**d**) Assessing liver function capacity after intravenous injection of ^13^C-methacetin by continuous measurement of ^13^CO_2_/^12^CO_2_-ratio: metabolism of the substrate in the liver by CYP1A2 enzymes forms ^13^CO_2_, which can be detected in exhaled breath. Figures created with BioRender.com.

Although exogenous compounds present extraneous confounders in breath studies that target endogenous biomarkers, conversely they represent an opportunity for assessing environmental exposure and pharmacokinetics [139,144]. Observations of their reduced levels in breath post-uptake as a result of metabolism, for example, might be as equally insightful as finding raised levels [19]. Accordingly, exogenous VOCs from daily exposure or through active introduction to induce a perturbation can be used to assess specific (disease-associated) processes or to identify population subgroups [144,145]. Several breath tests exist that exploit exogenous compounds, and all of these make use of the ingestion route. One such breath test, which does not target disease per se, is the well-known and widely implemented breath alcohol ‘breathalyser’ test, as used in law enforcement to identify drink-drivers [146]. Other targeted tests focus on the analysis of metabolic by-products from administered substrates, including the breath tests for hypolactasia [147], *Helicobacter pylori* infection [148], gastroparesis [149] and liver function [150] (depicted in Figure 5a–d). This section presents a discourse on the current breath tests that exploit exogenous substances or substrates for various means, commencing with the most common approach, before discussing the more specialised niche procedures.

### 4.1. Breath Alcohol Testing in Law Enforcement

The (over-)consumption of alcohol impairs the cognitive and psychomotor functions of the body, which has adverse effects on the execution of skilled tasks, such as driving a motor vehicle. Due to its physicochemical properties and related pharmacokinetics, ethanol can be readily detected in exhaled breath after alcohol consumption. The concentrations of alcohol in the blood and breath are highly correlated, which is utilised by law enforcement worldwide to identify drivers suspected of driving under the influence (DUI) of alcohol. Next to capnography, the breath alcohol test in law enforcement—colloquially known as the breathalyser test—is the most well-established and widely implemented breath-borne biomarker test [146,151]. 

The first breath alcohol analysis dates back to the mid-1860s, when British physician Francis Anstie made the first quantitative measurement of ethanol exhaled in human breath [152]. Using a breath trap containing chromic acid, which shifts in hue from red-brown to green in the presence of ethanol, Anstie demonstrated that only a small fraction of the consumed alcohol could be recovered in exhaled air. This led to the conclusion that the majority of the alcohol is metabolised in the body. 

**Figure 6:**
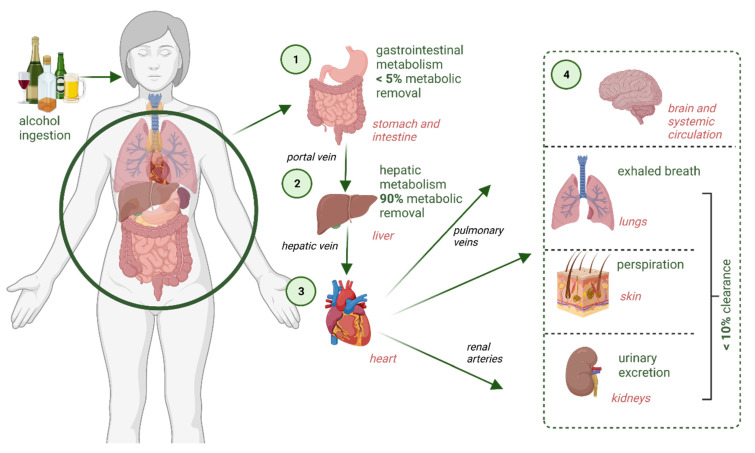
Alcohol (ethanol) disposition in the body. Only a small fraction (<10%) is eliminated unchanged through exhaled breath, perspiration and urinary excretion. Created with BioRender.com.

After alcohol is ingested, it is absorbed from the gut and transported to the liver, where approximately 90% is metabolised by oxidation with alcohol dehydrogenase (ADH) enzymes. Studies have shown that a maximum of 10% of the dose ingested is ultimately excreted unaltered via breath, urine and/or perspiration (Figure 6) [153,154,155].

Extensive studies have shown that the breath alcohol concentration (BrAC) is highly correlated with the concentration of alcohol in arterial blood circulation (blood alcohol concentration, BAC) over a wide range of ethanol dosages [156,157]. The elimination rates from the body are similar in both media [158]; this relationship demonstrates that BrAC is a suitable proxy to determine BAC [159]. This gives both the scientific and physiological premise for using an ethanol breath test for alcohol intoxication in law enforcement. 

Despite an excellent statistical correlation, however, there are certain limitations to the alcohol breath test in forensic practice, for example, samples should not be taken within at least 15 min after the end of drinking to avoid falsely overestimating BrAC (and, correspondingly, BAC) as a result of residual, unabsorbed ethanol present in the mouth [160,161]. Furthermore, a person’s breathing pattern, the temperature of the breath and the exhaled volume impact on the resulting BrAC [162,163]. The phase of ethanol metabolism also warrants consideration, as does specific health conditions, such as pulmonary functions [164,165]. These limitations with breath alcohol testing are well known and can be minimised to a large extent by following certain recommendations for quality assurance and, if necessary, the analysis of ethanol in alternative biological matrices, such as blood or urine, in some circumstances [160].

Diverse breath alcohol testing systems have been developed since the first devices emerged in the 1930s–1950s [166], transitioning from slow, manually operated wet-chemical instruments, incorporated in the Drunkometer [167] or the first Breathalyzer [168], to the more compact and easily operated infrared spectroscopy-based systems [146]. The current state-of-the-art breath alcohol instrumentation offers contact-free breath sampling (Servotek AB, Arlöv, Sweden) and exhibits an accuracy of >97% and a precision (coefficient of variation) of <1% [153,169,170]. Moreover, these contemporary instruments do not require a mouthpiece to be attached, because they simultaneously measure the exhaled water vapour concentrations, and the ethanol content is then standardised to the fully saturated water vapour concentration of alveolar air at body temperature [171]. These technical advances, together with empirical data on the relationship between BrAC and BAC, have led to widespread acceptance of breath alcohol analysis for legal purposes, with handheld devices for screening driver sobriety at the roadside, to evidential quality instruments, the results from which are used in criminal prosecutions [146,172,173]. Furthermore, also available are small and compact devices for use by the general population for self-control of sobriety before driving. 

The implementation of BrAC screening for a suspected DUI—with subsequent confirmatory BAC analyses—dates back to the 1950s, with the development of the first commercial Breathalyzer instrument, which became accepted and used in Australia, Canada and the USA [153]. Three decades later and extensive research established a quantitative relationship between BrAC and BAC and the worldwide acceptance of breath alcohol test results for evidential purposes. Instead of converting BrAC into BAC, however, most countries adopted a statutory BrAC limit, above which it has been made an offence to drive [153,174]. 

The legal permissible BrAC limits vary between countries and currently range from 0.10 to 0.40 mg/L, which equates to between approximately 0.2 and 0.8 g/kg (0.2–0.8‰) BAC [153]. The approval of use for breath alcohol devices in law enforcement varies depending on the country; in the USA, approval is granted by the National Highway Traffic Safety Administration of the US Department of Transportation. In relation to FDA approval, the first approved device for breath ethanol testing was the AlcoMate CA2000 Digital Alcohol semiconductor oxide sensor detector (KHN Solutions LLC, San Francisco, CA, USA), issued in 2004, with various other devices being approved since then [29]. Today, breath alcohol tests are in widespread and frequent use worldwide. The ‘breathalyser’ test does not represent a disease diagnostic procedure, yet it demonstrates the high utility of a breath-based approach and its potential for practical applications beyond healthcare. The breath alcohol test is the most common breath-borne biomarker-based procedure that is encountered in everyday life. 

### 4.2. Hydrogen Breath Test in Hypolactasia

Lactose intolerance, or hypolactasia, is a condition in which lactose cannot be readily digested due to insufficient lactase enzymes in the gut. Specifically, the malabsorption of this sugar is caused by a reduced expression of lactase in the small intestine, leading to patient discomfort with abdominal complaints, such as diarrhoea, flatulence or pain [175,176]. Several clinical tests exist to diagnose lactose malabsorption [177], including the measurement of serum glucose, genetic tests and breath testing [178,179,180]. In the past, a lactose activity assay by jejunal biopsy was proposed as the gold standard [175,181], but this has been superseded by the non-invasive hydrogen breath test (HBT), which is one of the few breath-based approaches that is applied for routine clinical purposes and the most widely used method for the determination of lactose malabsorption [182,183,184]. 

Hydrogen breath tests are based on the principle that hydrogen gas is not endogenously produced other than by intestinal bacteria as they metabolise carbohydrates, such as lactose, that are insufficiently absorbed in the colon; accordingly, the HBT can be considered a biological/non-host-driven test (see Section 2). Following its production by intestinal bacteria, intracolonic hydrogen (H_2_) diffuses through the colon wall into the systemic circulation and is carried to the lungs, where it is excreted from the body via exhaled breath (Figure 5a) [182,185,186,187]. 

As the ingested substrate (lactose) is considered a common ingredient of food consumed in large quantities on a daily basis, the HBT itself is exempt from approval by the FDA; although, the first HBT instrument, Micro H2, using sensor technology (Micro direct, Inc., ME, USA), received FDA approval in 1997, followed by other HBT testing devices in subsequent years [29]. Various techniques have been put forward to perform the HBT, which differ according to the type of substrate (lactose or milk), its quantity (physiological or tolerance test), and the duration of the test (one to six hours) and its sampling intervals [181,182,188,189,190]. Nowadays, the HBT involves an oral challenge with a standardised dose of lactose (usually 20–50 g, but up to 100 g, corresponding to a lactose content of approximately 400–2000 mL bovine milk), with subsequent monitoring of exhaled H_2_, usually over a period of about two hours; lactose intolerance is then determined when breath H_2_ remains at significantly elevated concentrations within the respective test period (cut-off value > 20 ppm) [147,175,180,183]. 

Although HBTs are in routine use in clinical practice, the interpretation of the test results can be challenging due to several factors that influence its accuracy. The premise of the HBT is the presence of hydrogen-producing bacteria, yet there is a considerable proportion of patients in whom the colonic flora does not produce hydrogen, referred to as ‘H_2_-non-producers’, which leads to false-negative results [180,186,191]. Another possible cause for false-negative results could be a full colonic adaptation to lactose ingestion due to the favoured growth of lactose metabolising bacteria without hydrogen production [192,193]. Usually, patients produce either H_2_ or methane gas (CH_4_); however, about 30% of the adult population possess methanogens that produce methane at the expense of hydrogen in the gut. In these cases, complementary CH_4_ detection in breath can enhance the HBT and, therefore, might improve the correct diagnosis of malabsorption issues [194,195]. Hydrogen-methane breath testing has been hitherto underutilised due to the lack of low-cost, easy-to-operate instruments, but recent technical advances have led to the emergence of commercial devices [194], such as the BreathTracker^TM^ SC analyser by QuinTron Instrument Company, Inc. (Milwaukee, WI, USA), which separates the compounds in an alveolar gas sample using a fast GC combined with a solid-state sensor and is used to identify carbohydrate malabsorption and small intestinal bacterial overgrowth (SIBO) sufferers [196]. Other devices target multiple components by incorporating electrochemical H_2_ sensors and IR sensors for CH_4_ and CO_2_ detection (Lactotest 202 by Medical Electrionic Construction, Nivelles, Belgium) [197], or IR sensors for the simultaneous measurement of H_2_, CH_4_ and O_2_, with additional electrochemical sensors for CH_4_ and H_2_ detection (GastroCH_4_ECK by Bedfont Scientific Ltd., Maidstone, UK) [195]. Methane breath testing is, therefore, an emerging and promising complement to the HBT. An additional potential source of error in the HBT is carbohydrate malabsorption in relation to chronic pancreatitis and coeliac disease [198,199]. Conversely, false positives can arise due to smoking, oral bacterial flora or SIBO, or from a high intake of dietary fibres on the day prior to testing [180,185,200]. Accordingly, tracking the nutritional history and symptoms of patients, as well as measuring the blood glucose levels and exhaled methane, are helpful in correctly interpreting HBT results [178,183]. 

### 4.3. ^13^C-Breath Tests in Clinical Applications

Elemental carbon naturally occurs in three isotopes, with ^12^C and ^13^C being stable. In the past, breath tests were developed using the radioactive isotope ^14^C for testing exocrine pancreatic function; however, its radiation hazard and long half-life (5730 years) led to the increasing use of ^13^C as a substitute for diagnostic tests [148,201]. In 1973, Lacroix and co-workers first reported the use of ^13^C as a tracer compound in a human metabolomics study, whereby the abundance of exhaled ^13^CO_2_ was followed after the ingestion of ^13^C-labelled glucose [202]. Nowadays, ^13^CO_2_ is used in several diagnostic tests as an in vivo biomarker in exhaled breath after ingesting specific ^13^C-substrates, pushing the way forward in personalised medicine [83]. In contrast to the HBT, the field of application of ^13^CO_2_ breath tests is wider due to the diversity in available substrates. The choice of the substrate determines the target of the respective breath test, from potential gastric bacterial *Helicobacter pylori* infection, gastric emptying, liver and pancreatic function, to the assessment of other enzyme activities. The common basis of these breath tests is the use of ^13^C-labelled tracer probes that undergo metabolism via a pathway of interest and produce ^13^CO_2_ as a metabolite, which is subsequently expelled through respiration and detectable in exhaled breath [203]. By monitoring the unidirectional decomposition to ^13^CO_2_, the turnover of the substrate can be assessed; this is based on the premise that the process under investigation is driving the excretion rate of ^13^CO_2_, since other metabolic processes are negligibly fast or not variable. It is worth noting that the tracer compound cannot be fully recovered in breath, since a part of it is stored in the carbon pool of the human body; thus, such approaches are only semi-quantitative [186]. Despite substantial research in ^13^CO_2_-based breath testing, only three tests are currently approved by the FDA/EMA, namely the ^13^C-urea breath test (UBT) for *Helicobacter pylori* infection, ^13^C-*spirulina* for gastroparesis, and ^13^C-methacetin for liver maximum (LiMax) function assessment [203], which are presented in the following sections.

#### 4.3.1. ^13^C-Urea Breath Test for Diagnosis of Helicobacter Pylori Infection

The most prominent application of stable isotope substrates and subsequent ^13^CO_2_ monitoring is the ^13^C-urea breath test (UBT), which detects a *Helicobacter pylori* (*H. pylori*) infection in the gut. *H. pylori* is a common bacterium infecting at least 50% of the world’s population [204,205]. The microbe produces large amounts of the enzyme urease, which hydrolyses urea in the stomach [148]. Even though the majority of infected people are asymptomatic, an *H. pylori* infection has been associated with different diseases, such as peptic ulcer disease, non-ulcer dyspepsia and gastric cancer [206,207,208]. The close association of an *H. pylori* infection with gastric cancer has led to the classification of this bacterium by the World Health Organization (WHO) as a group 1 carcinogen [209,210]. The assessment of possible infection is, therefore, crucial to enable suitable antibiotic therapy.

The UBT has undergone several evolutions since its first reported use in 1987 [211], namely adjustments in the test protocol relating to the fasting state, the type and quantity of the substrate and the sampling intervals [212,213,214,215]. Generally in this test, orally administered ^13^C-urea is hydrolysed by the bacterial urease activity in the stomach to form ^13^C-labelled CO_2_ (and ammonia, NH_3_), which is absorbed through the mucus layer of the stomach, transported to the lungs with the bloodstream and excreted via exhaled breath (Figure 5b) [148]. After an initial baseline breath sample collection, a patient will typically ingest 75 mg of ^13^C-urea and will provide a second breath sample 20 min later. An enrichment in exhaled ^13^CO_2_ of >2.4‰, or about 26 ppm, as determined with isotope ratio mass spectrometry, is evidence of an active infection, which is expressed as delta over baseline (DOB) [203]. 

Various factors suppressing bacterial growth can affect the test results, including the intake of proton pump inhibitors, H_2_ antagonists or antibiotics, which can reduce sensitivity and might cause false negative results [186,216]. Furthermore, it is common practice for patients to receive a standardised test meal to ensure a consistent diagnostic accuracy of the UBT for pre- and post-treatment for an *H. pylori* infection. Multiple studies and a meta-analysis that included more than 3500 patients reported the high sensitivity (>95%) and specificity (>95%) of the UBT compared to the histology [212,217,218,219]. Its accuracy has been compared to the invasive diagnostic rapid urease test, histology and culture after a gastric biopsy during an endoscopy [220] and the non-invasive faecal antigen test [208], and it has been claimed to be superior to the previous methods [186].

In the past, an *H. pylori* infection was diagnosed by a non-invasive stool antigen test and serology, or via invasive means, such as histology, rapid urease test, and culture as biopsy-based endoscopic tests [201,221]. Since its approval by the FDA in 1996, the non-invasive UBT has been instrumental in reducing endoscopic procedures (and the associated costs) in clinical screenings. A commercial UBT kit for the diagnosis of an *H. pylori* infection was launched on the market in 1997 by Meretek Diagnostics Inc. (Lafayette, CO, USA), followed by the approval of additional UBT devices, such as the Exalenz BreathID Hp System (Exalenz Biosciences Ltd., Modi’in, Israel) in 2011, or more recently, the successful premarket approval in 2020 for the PyloPlus UBT system (ARJ Medical, Oldsmar, FL, USA) [221].

#### 4.3.2. Gastric Emptying Breath Test for Gastroparesis

Gastroparesis is a muscular disorder that delays the gastric emptying of food, commonly resulting in symptoms of early satiety, postprandial fullness, nausea, vomiting, belching and bloating. Since the condition is part of a catalogue of gastric neuromuscular dysfunctions with overlapping symptoms, it is essential to be able to distinguish between them [222]. In 1833, Beaumont first observed the emptying of gastric contents through a gastric fistula [223]. Since then, various methods have been developed to gauge the degree of gastric emptying, but no single technique has the ability to describe comprehensively the gastrointestinal transit after the ingestion of a heterogeneous meal comprising solid and liquid components [224]. This shortcoming relates to the distinctive emptying between solids and liquids, smaller and larger solid particles, and the lipid and aqueous phases of gastric contents [225]. Scintigraphy currently represents the gold standard reference method for determining the gastric emptying of solid and liquid meals [226,227]. The procedure, which must be executed by highly qualified staff, involves the use of a test meal that incorporates potentially harmful radioactive isotopes; thus, alternative methods are highly desirable [224,228,229]. The gastric emptying breath test (GEBT) is a comparatively safe technique to assess digestive function and provides a reliable diagnosis of gastroparesis [149]. In contrast to scintigraphy, the GEBT makes use of ^13^C-substrates, either ^13^C-octanoic acid [230] or ^13^C-enriched *Spirulina platensis* (*S. platensis*) (an edible blue-green alga) for solid gastric emptying, or ^13^C-sodium acetate for liquid gastric emptying [231], all of which are suitable and safe for critically ill patients, pregnant women and children [201,221]. These substrates are quickly absorbed in the proximal small intestines and are subsequently metabolised by the liver, leading to the production and ultimately excretion of ^13^CO_2_, which is eliminated from the body via respiration (Figure 5c). The time limiting step from substrate ingestion to the appearance of elevated ^13^CO_2_ concentrations in the breath provides a measure for the rate of gastric emptying [232], although the detection of alterations in the gastric emptying of solids is generally more sensitive than for liquids [233]. 

The GEBT procedure requires three to four hours, whereby breath samples are collected once pre-ingestion and at five to six intervals post-ingestion of the test meal labelled with the ^13^C substrate, such as ^13^C-*spirulina* [221]. A post-meal enrichment of ^13^C in relation to ^12^C is determined in exhaled CO_2_ using isotope ratio mass spectrometry, which allows the rate change of ^13^C in the breath (in µmol/L/min) to be calculated using the DOB approach [234]. A suppression of gastric emptying is indicated through a delayed increase in ^13^CO_2_ and a subsequent slower recovery rate of ^12^CO_2_, and measures of these phenomena are then used to calculate the gastric emptying half-time and lag phase duration [228], which reflect the overall emptying functions and the ability of the stomach to triturate solid food to smaller particles that can be emptied [235]. 

Despite the aforementioned benefits, the GEBT is not widely implemented as an alternative to scintigraphy, largely due to a lack of awareness of this approach by physicians and patients. Nevertheless, validation studies have demonstrated that the GEBT has a specificity and sensitivity of 89–98% and 24–64%, respectively, with positive predictive values of 73–97% and negative predictive values of 65–90%, depending on the chosen time point for diagnosis [236]. In 1981, the FDA acknowledged ^13^C-*S. platensis* as a ‘legally marketed’ food with health benefits due to its high nutritional qualities, thereby bypassing the need for specific approval for the adminstration of the substrate. The GEBT for solids using this algae, developed by Cairn Diagnostics (Brentwood, TN, USA), received FDA approval in 2015 [221]. The GEBT is a validated and standardised procedure that has been demonstrated to be highly reliable compared to the gold standard method, thus representing a suitable, non-invasive alternative to the hitherto established diagnostic approach for gastroparesis.

#### 4.3.3. Maximum Liver Function Capacity Breath Test

Hepatologists have been studying potential ^13^C-breath tests to examine the cytosolic, mitochondrial and microsomal hepatic function associated with various liver diseases for over four decades [203,237]. In contemporary medicine, hepatectomy represents the treatment of choice for liver malignancies, yet the procedure carries a significant risk of post-operative liver failure—and death—following the resection of the hepatic tissue, a risk that could be lowered through knowledge on the pre- and post-surgery liver function capacity [238,239]. Various tests to evaluate liver dysfunction exist, including methods based on protein synthesis in the liver (e.g., prothrombin, albumin), hepatocellular integrity (transaminases), cholestasis and excretion (bilirubin, alkaline phosphatase, gamma-glutamyltransferase), as well as other techniques [240]. In addition, scoring indices for assessing liver damage severity and monitoring patients, such as the model for end-stage liver disease (MELD) and Child–Turcotte–Pugh (CTP), are widely used. A major shortcoming of these tests, however, is that they only provide an assessment of liver dysfunction (or injury) rather than function. Due to the capacity of the liver to regrow and rejuvenate, it is critical for haematologists to evaluate function rather than injury or dysfunction, since even a compromised liver can exhibit sufficient functionality. Further, on the one hand, there is a need to evaluate the liver function of patients undergoing a hepatic resection in order to identify eligible candidates for liver transplantation [241], and on the other hand, the early detection of post-operative liver failure is essential [239,242].

Breath-based tests that utilise ^13^C-labelled substances have been examined for different applications, from evaluating the residual liver functional capacity or function after liver transplantation, to assessing the severity of liver fibrosis from early stages up to liver cirrhosis [201,243]. A variety of ^13^C-labelled substrates has been studied for their use in assessing hepatic function, including ^13^C-phenylalanine, ^13^C-aminopyrine, ^13^C-erythromycin and other compounds [201,244,245,246,247]. One specific substrate is the paracetamol prodrug ^13^C-methacetin, which targets the CYP450-dependent enzymatic system and is used to determine maximum liver capacity, or LiMAx [150,221]. ^13^C-Methacetin is exclusively metabolised by CYP450 1A2 (CYP1A2), whereby the cleaved methyl group is oxidised to formic acid, before entering the C1 pool and subsequently exhaled as ^13^CO_2_ (Figure 5d) [186,248]. The enzyme is not influenced by genetic variations or drugs and is ubiquitous in the liver, making it the ideal target to assess liver function capacity [239]. 

As with the ^13^C-based tests discussed above, the LiMAx breath test involves determining the patient’s DOB via an initial measurement of the ^13^CO_2_/^12^CO_2_ ratio. ^13^C-Methacetin is then administered intravenously at a body-weight-adjusted quantity and the ^13^CO_2_/^12^CO_2_ ratio in the breath—which changes as a function of liver capacity—is monitored in real-time over the course of an hour [248]. Liver function is classified into three levels based on the metabolising capacity of CYP1A2, as determined from the latter ratio, namely normal liver function, with a lower cut-off of 315 µg/kg/h, intermediate liver function, with 140–315 µg/kg/h, and strongly impaired hepatic function, with a LiMAx < 140 µg/kg/h. The LiMAx test has been demonstrated to be highly reproducible in subjects with normal liver function, with a correlation coefficient of the repeat LiMAx test of 0.85 (95% confidence interval 0.69–0.93) [239]; further, age, sex and obesity have not been observed to influence the test. Due to the invasive (intravenous) nature of the substrate administration, the LiMAx test has yet to receive FDA approval and its implementation in the US market is challenging, yet an EMA approval of the test in 2017/2018 for its clinical use in Germany, Austria and the United Kingdom [221] has led to its current practice in clinical diagnostics in more than 20 hospitals in Europe [248]. 

## 5. Summary and Outlook

Metabolomics is an emerging discipline that offers a promising opportunity to examine the human physiology in relation to different scenarios, from screening for disease, to targeting drug metabolism, or estimating the burden of environmental stressors; it thereby serves as a potential tool for objective diagnostics. In comparison to other metabolic profiling approaches, the analysis of human exhaled breath represents a dynamic and multifaceted technique with high prospective value. The benefits of breath-based tests are indisputable: breath can be provided non-invasively, on demand and repeatedly, offering patient comfort, flexibility and avoiding the specific need for privacy or highly skilled personnel; the analysis of certain gas-phase biomarkers can be performed directly (online) and deliver immediate results; further, aside from airborne pathogens, exhaled breath is typically non-infectious and does not generate hazardous waste. Despite these benefits, breath analysis has its limitations: most gas-phase volatile chemical compounds are present in the breath at ultra-trace concentrations and exhibit a degree of lability, making many compounds challenging to sample efficiently and analyse accurately; their high qualitative and quantitative variability, their commonality in different diseases, and their ubiquity in the environment present further challenges in identifying representative, disease-specific biomarkers. 

Nevertheless, several breath-based tests have emerged to become established in clinical practice or other settings. These tests vary in their approach in targeting compounds that are either of endogenous or exogenous origin. Beyond the breath-based tests reviewed here, current breath research is exploring several promising avenues. Notably, breath analysis has received unprecedented attention recently in relation to the severe acute respiratory syndrome coronavirus-2 (SARS-CoV-2) outbreak and the associated coronavirus disease 2019 (COVID-19) pandemic. The potential for exhaled breath to either detect this airborne virus directly or to diagnose infection is currently being investigated as a comfortable alternative to existing approaches that collect mucus secretions via nasopharyngeal or oropharyngeal swabs, or serological samples [249,250,251]. While no breath test has yet been developed that allows a reliable detection of the infection, studies have reported potential breath-borne VOC biomarkers (detected via gas chromatography ion mobility spectrometry, GC-IMS) [252] or specific breathprints (using proton transfer reaction time-of-flight mass spectrometry, PTR-TOF-MS) [253] for COVID-19, as well as evidence for tests using nanomaterial-based sensors [254]. Further, although not treated in this review, exhaled breath condensate (EBC) has been explored as an alternative medium to mucus collected by nasopharyngeal swabs to detect the presence of COVID-19 via reverse transcriptase-polymerase chain reaction (RT-PCR) [255]. There is much research currently being undertaken on detecting COVID-19 in exhaled breath, and the data collected from early pilot studies suggest that a non-invasive breath test is a viable future approach to screening for infection. 

Besides COVID-19, breath testing has a promising future in other areas of application. One particularly innovative approach relates to the potential diagnosis of liver cirrhosis in relation to exhaled limonene. Limonene in the breath is widely accepted to be of exogenous origin, deriving primarily from the diet (e.g., being present at high concentrations in citrus fruits and juices), or to a lesser extent through inhalation due to its ubiquitous presence in the environment. In a 2015 study that investigated the exhaled breath composition of patients with a cirrhotic liver, it was observed that concentrations of limonene (as well as methanol and 2-pentanone) in the patient group were significantly elevated compared to healthy controls [256]. These observations were explained by the inability of the compromised liver to breakdown this exogenous compound, compared with its efficient metabolism by CYP450 enzymes in the healthy cohort. Follow-up studies have further explored this phenomenon, with the inclusion of patient groups with hepatocellular carcinoma (HCC) and/or hepatic encephalography (HE) [257,258]. Similar observations were made, although no differences were found between liver cirrhosis with or without HCC, and the degree of HE severity could not be linked to limonene concentrations, although limonene accumulation was speculated to be itself a causative pathway for HE. The innovative aspect of these first pilot studies is that they exploit a dietary/environmental constituent that is not expressly administered to represent a marker of organ function, which is a hitherto unexplored area of breath research. Further, these studies serve as the basis for the development of tests using defined substrates—exogenous VOC (EVOC) probes—to examine the metabolic breakdown of a target compound to ascertain compromised enzymatic activity. This approach is currently the subject of investigation [145].

Looking at the established and/or approved breath tests, as discussed in this review, three of the eight tests make use of ^13^C-labelled substrates, which underlines the potential of such targeted approaches in breath analysis. Indeed, it can be expected that different ^13^C-labelled tracer probes will join the collection of breath tests for clinical routine purpose in future. The current developments that focus on the analysis of ^13^CO_2_ include a test that utilises an oral administration of ^13^C-methacetin as a substrate as opposed to intravenous administration (see Section 4.3.3; Figure 5d) to assess metabolism and liver capacity, a breath-based glucose tolerance test using ^13^C-labelled glucose, and a substrate-based bacterial overgrowth assessment, as well as a pancreatic inefficiency test, as reviewed in the literature [221]. Further, numerous studies have observed associations between breath volatiles and sputum or blood inflammatory cells, which highlight the prospects of breathomics as a future clinical tool for disease phenotyping and personalised medicine [259,260,261]. 

A key lesson learnt from the F_E_NO test for asthma is that standardisation is essential to establish a reliable, reproducible and meaningful test. The lack of standardised practices in breath research has been a major impediment to progress in the field [11]. Accordingly, the current initiatives on standardisation and benchmarking are worthy of mention here. Specifically, the *International Association of Breath Research* (IABR) has instigated a pilot study to explore the establishment of a benchmarking protocol for the measurement of VOCs in the breath in order to allow for comparison between sampling and analysis approaches. The ‘Peppermint Experiment’ is a pharmacokinetic-based undertaking that focusses on the release and excretion of volatile peppermint oil constituents after the ingestion of a capsule containing the oil [262]. In short, measuring the washout curves of the specified compounds in exhaled breath over a defined timeframe allow for mean washout times to be calculated for any one sampling and/or analytical approach, allowing for comparison with other datasets. The consortium of participating research laboratories currently numbers 16 from seven countries, with the initial pilot studies comprising 1200 breath samples collected from 200 participants, and with analyses performed using GC-MS [263,264], secondary electrospray ionisation mass spectrometry (SESI-MS) [264,265], as well as PTR-MS and selected ion flow tube-mass spectrometry (SIFT-MS) [266], and with the publication of datasets from other approaches, such as GC-IMS, pending. Although the comprehensive datasets from these feasibility studies have highlighted the necessity to improve and refine the experimental protocol, the approach taken represents a first concerted effort within the broader breath analysis community to establish a method to allow for quality assurance checks of breath data. Ultimately, a compartmentalised approach to standardising practices in breath sampling and analysis is needed in order to cater for the broad spectrum of methods and target diseases [12].

The existing breath tests reviewed in this paper demonstrate the efficacy of breath-based diagnostics. Breath analysis remains an innovative and compelling approach for companion diagnostics and personalised medicine. Continual advancements in sampling approaches and analytical technologies, as well as data mining tools, will serve to generate cogent evidence for breath-based testing. The field holds much promise, especially for intervention-based approaches or personalised monitoring, but for new breath tests to succeed and transition to routine applications, the corresponding data must be cross-validated and exhibit high sensitivity, selectivity and accuracy, as well as offer a benefit over conventional diagnostic approaches.

## Figures and Tables

**Table 1:** Current established/approved breath-based tests that utilise endogenous or exogenous gas-phase compounds.

	Application or Disease Target	Test Name	Target Compound	Detection Method	Unit of Measurement	Determinant and Setting * of Use
Endogenous compounds	Ventilation/breathing	Capnography	Carbon dioxide (CO_2_)	Colorimetric CO_2_ detector, mainstream and sidestream CO_2_ monitoring (IR spectroscopy)	mmHg	Routine, clinical
	Asthma	F_E_NO	(Fraction of exhaled) nitric oxide (NO)	Chemiluminescence analyser, electrochemical sensors and laser-based technology	ppb	Symptomatic, clinical/surgery
	Neonatal jaundice	CO	Carbon monoxide (CO)	CO monitor with integrated IR breathing sensor and electrochemical sensor	ppm	Symptomatic, clinical
	Grade 3 heart transplant rejection	Heartsbreath	Alkanes	TD-GC-MS	Breath methylated alkane contour	Targeted, clinical
Exogenous compounds	Alcohol intake	Breath alcohol test (Breathalyser)	Ethanol (CH_3_CH_2_OH)	IR spectroscopy, electrochemical fuel cells, dual sensor devices (electrochemical oxidation and IR absorption)	mg/L, ‰	Targeted, mobile
	Lactase deficiency	Hydrogen breath test	Hydrogen (H_2_)	Hydrogen breath analyser with integrated electrochemical gas sensor	ppm	Symptomatic/targeted, surgery
	*Helicobacter pylori* infection	Urea breath test (UBT)	^13^CO_2_	Isotope ratio mass spectrometry	ppm, ‰	Symptomatic/targeted, surgery
	Gastroparesis	Gastric emptying breath test (GEBT)	^13^CO_2_	Isotope ratio mass spectrometry	µmol/L/min	Symptomatic/targeted, surgery
	Liver function	Maximum liver function capacity (LiMAx)	^13^CO_2_	Isotope ratio mass spectrometry	µg/kg/h	Targeted, surgery

* Settings referred to are *clinical*, i.e., in-patients in hospitals or medical centres; *surgery*, i.e., ambulant patients in the physician’s practice (or medical centres); *mobile*, i.e., in the field or in specific settings (only applicable for the breath alcohol test). Abbreviations: IR = infrared; TD-GC-MS = thermal desorption-gas chromatography-mass spectrometry.
